# TRPM3-Induced Gene Transcription Is under Epigenetic Control

**DOI:** 10.3390/ph15070846

**Published:** 2022-07-10

**Authors:** Gerald Thiel, Oliver G. Rössler

**Affiliations:** Department of Medical Biochemistry and Molecular Biology, Saarland University, Building 44, 66421 Homburg, Germany; oliver.roessler@uks.eu

**Keywords:** AP-1, A485, BET proteins, bromodomain, Elk-1, interleukin-8, JQ1, CREB, TRPM3

## Abstract

Transient receptor potential M3 (TRPM3) cation channels regulate numerous biological functions, including gene transcription. Stimulation of TRPM3 channels with pregnenolone sulfate activates stimulus-responsive transcription factors, which bind to short cognate sequences in the promoters of their target genes. In addition, coregulator proteins are involved that convert the chromatin into a configuration that is permissive for gene transcription. In this study, we determined whether TRPM3-induced gene transcription requires coactivators that change the acetylation pattern of histones. We used compound A485, a specific inhibitor of the histone acetyltransferases CBP and p300. In addition, the role of bromodomain proteins that bind to acetylated lysine residues of histones was analyzed. We used JQ1, an inhibitor of bromodomain and extra terminal domain (BET) family proteins. The results show that both compounds attenuated the activation of AP-1 and CREB-regulated gene transcription following stimulation of TRPM3 channels. Inhibition of CBP/p300 and BET proteins additionally reduced the transcriptional activation potential of the transcription factors c-Fos and Elk-1. Transcriptional upregulation of the interleukin-8 gene was attenuated by A485 and JQ1, indicating that proinflammatory cytokine expression is controlled by CBP/p300 and bromodomain proteins. We conclude that TRPM3-induced signaling involves transcriptional coactivators and acetyl-lysine-bound bromodomain proteins for activating gene transcription.

## 1. Introduction

TRPM3 (Transient receptor potential M3) channels are nonselective cation channels that can be activated with the neurosteroid pregnenolone sulfate [1,2]. Numerous biological functions are associated with TRPM3 activation, including temperature and pain sensation, insulin secretion, contraction of vascular smooth muscles, and tumorigenesis [3]. In the somatosensory system, TRPM3 functions as a thermosensor and chemosensor. Analysis of transgenic mice that did not express TRPM3 revealed that the animals did not response adequately to noxious heat [4]. Genetic experiments revealed that perception of acute noxious heat sensing requires TRPM3, as well as TRPA1 and TRPV1 [5]. In addition, transcription mediated by AP-1, c-Jun, c-Fos, Elk-1 and Egr-1 is induced as a result of TRPM3 stimulation [2,3,6]. These stimulus-responsive transcription factors interact with the regulatory regions of their target genes, resulting in enhanced transcription. The gene products of these target genes contribute to the physiological and biochemical alterations observed after TRPM3 stimulation. We have recently shown that TRPM3-induced activation AP-1 stimulates transcription of the delayed response gene encoding the cytokine interleukin-8 [7]. The expression of interleukin-8 could lead to inflammatory changes as a consequence of TRPM3 stimulation.

The signaling cascade triggered by stimulation of TRPM3 channels leads to binding of stimulus-activated transcription factors to short DNA sequences in the promoters of their target genes. In addition, transcription factors interact with numerous coregulator proteins to decompact repressive chromatin structures. Chromatin comprises DNA complexed with histone proteins. The DNA packaging in chromatin is essential for gene transcription because the chromatin can be compact so that transcriptional regulators cannot bind to the DNA, or it can have an open configuration that allows interactions between DNA and transcription factors and recruitment of RNA polymerase II to DNA. Transcriptional activators bind to various coregulator proteins that either function as chromatin-modifying enzymes or are part of ATP-dependent protein complexes that modulate the chromatin status. The architecture of the chromatin is modified by enzymes that transfer or remove post-translational modifications of histones. One important modification is acetylation [8,9]. Histone acetyltransferases catalyze the acetylation of lysine residues of histones 3 and 4. Acetylation of histones reduces positive charges, thereby reducing the interaction between DNA and histone proteins. As a result, chromatin converts to an open configuration favorable for gene transcription [10]. Moreover, acetylated histones are binding sites for numerous proteins that specifically interact through their bromodomain with acetylated histones [11,12]. Conversely, deacetylation of histones, catalyzed by histone deacetylases, leads to chromatin compaction, and this enzymatic reaction is connected with transcriptional repression.

Here, we investigated whether TRPM3-induced gene transcription depends on the recruitment and biological activity of coactivators that change the acetylation pattern of histones. We used a pharmacological approach to inhibit the histone acetyltransferase activities of the paralogs CBP (CREB-binding protein) and p300. Both proteins are master regulators of gene transcription that interact with numerous transcription factors and modify their activities [13,14,15]. In addition, we pharmacologically blocked bromodomain and extra terminal domain (BET) protein interactions with acetylated histones. The results show that epigenetic regulatory proteins such as CBP/p300 and BET proteins are required for TRPM3 channel-mediated gene transcription, indicating that TRPM3-induced transcription is under epigenetic control.

## 2. Results

### 2.1. CBP/p300 and Bromodomain and Extra-Terminal (BET) Inhibitors Attenuate TRPM3-Induced Activation of AP-1

**Figure 1:**
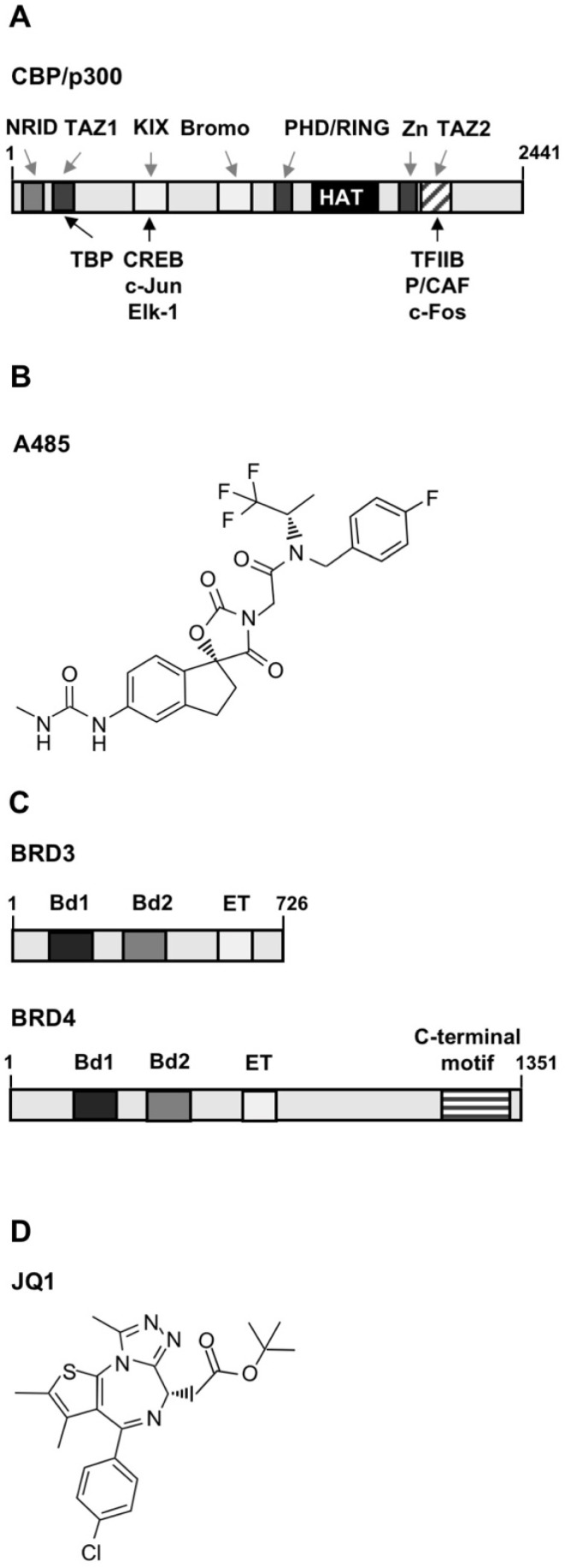
Pharmacological inhibitors of epigenetic gene regulation. (**A**) Modular structure of the transcriptional coactivator and acetyltransferase CBP. The paralog p300 has a similar modular structure. Both proteins have a nuclear receptor interaction domain (NRID), two transcriptional adapter zinc finger domains (TAZ), a kinase inducible domain interacting domain (KIX) that binds CREB, the CREB-related protein ATF-1, c-Jun and other transcription factors, a bromodomain, a PHD-RING module, and a histone acetyltransferase (HAT) domain. The C-terminal TAZ domain binds c-Fos. (**B**) Chemical structure of the CBP/p300 catalytic inhibitor A485. (**C**) Modular structure of the BET proteins BRD3 and BRD4. BET proteins have two bromodomains (BD) as well as an extra-terminal (ET) domain that functions as a protein binding module for other chromatin-modifying proteins. BRD4 contains additionally a C-terminal domain (CTD) that functions as a binding site for P-TEFb (positive transcription elongation factor b). (**D**) Chemical structure of the pan-BET-inhibitor JQ1.

Pharmacological and genetic experiments have shown that stimulation of TRPM3 induces transcription mediated by the transcription factor activator protein AP-1 [2,3,6]. We therefore investigated whether pharmacological inhibition of CBP/p300, and subsequent disruption of histone acetyltransferase activity, attenuates AP-1 activation as a result of TRPM3 channel stimulation. Figure 1A shows the modular structure of CBP.

**Figure 2:**
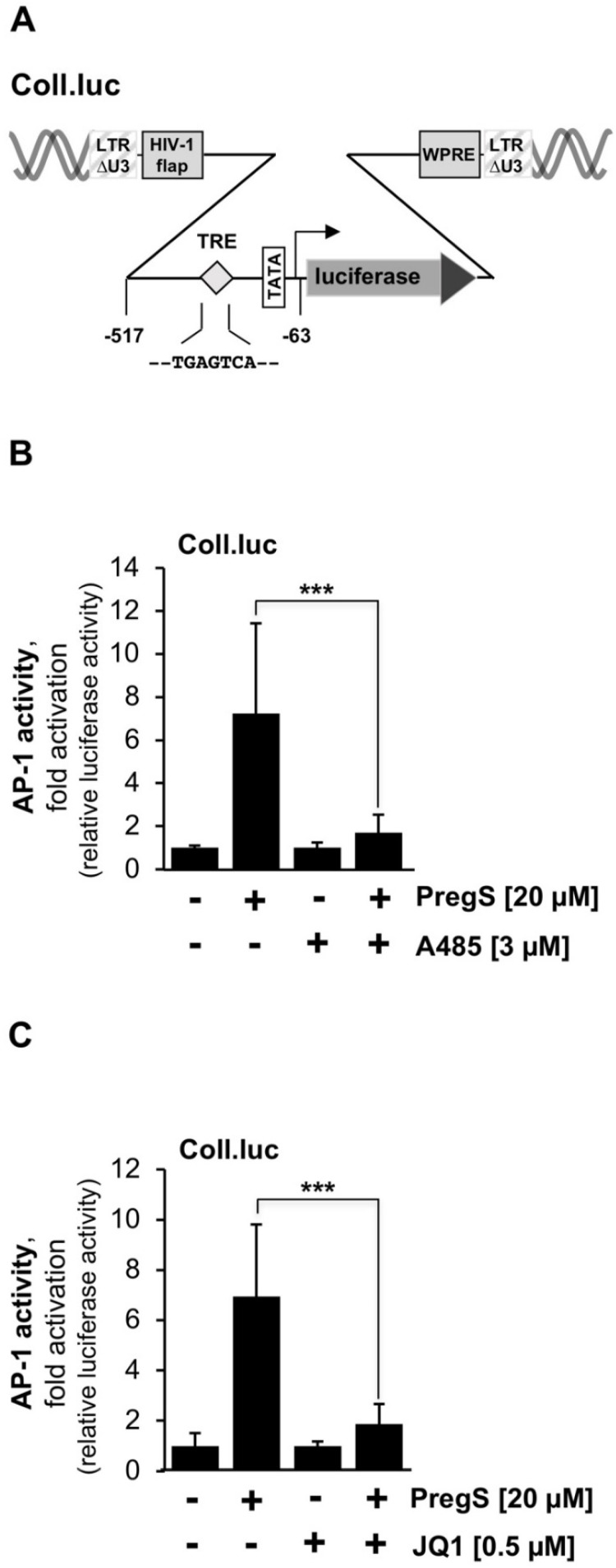
Pharmacological inhibition of CBP/p300 and BET proteins attenuates TRPM3-mediated activation of AP-1. (**A**) Provirus containing the Coll.luc (collagenase promoter/luciferase) reporter gene. The AP-1 DNA binding site (known as 12-*O*-tetradecanoylphorbol-13-acetate (TPA)-responsive element (TRE)) is indicated. The provirus contains a deletion within the 5′LTR U3 region. Additionally, the provirus contains the woodchuck hepatitis virus posttranscriptional regulatory element (WPRE) and the HIV flap element. (**B**,**C**) T-REx-TRPM3 cells were infected with a Coll.luc-containing recombinant lentivirus. Cells were incubated in a serum-reduced medium for 24 h that was supplemented with tetracycline (1 μg/mL) to induce TRPM3 expression. Preincubation of the cells was performed for 3 h with either the histone acetyltransferase inhibitor A485 (3 μM) (**B**), or the BET inhibitor JQ1 (0.5 μM) (**C**). T-REx-TRPM3 cells were stimulated for 24 h with pregnenolone sulfate (PregS, 20 μM) in the presence of the inhibitor. Cell extracts were prepared and analyzed for luciferase activities. Luciferase activity was normalized to the protein concentration. Data shown are mean +/− SD of four (**B**) or three (**C**) independent experiments performed in quadruplicate (*** *p* < 0.001).

There are several methods to measure stimulus-induced gene transcription, including measurement of nuclear translocation of transcription factors, measurement of transcription factor expression levels, phosphorylation, or DNA binding. The most reliable method to detect and quantify stimulus-induced transcription factor activation is to use a transcription factor-responsive reporter gene embedded in chromatin that allows direct measurement of transcriptional activation [16]. The reporter gene must be implanted into the chromatin of the cells to ensure that the reporter genes are packed into an ordered nucleosomal structure. The Coll.luc reporter gene was used as a sensor to measure AP-1 activity (Figure 2A) [16]. The reporter gene was integrated into the chromatin via lentiviral gene transfer. This approach was the prerequisite for analyzing epigenetic gene regulation following stimulation of TRPM3 channels. T-REx-TRPM3 cells were used as a cellular model system. In these cells, the addition of tetracycline to the culture medium induces TRPM3 biosynthesis, as shown previously [2]. TRPM3 channels are able to activate voltage-gated Ca^2+^ channels [3] which are not expressed in T-REx-TRPM3 cells [17]. We therefore avoided an interference between TRPM3 and voltage-gated Ca^2+^ channel. We infected T-REx-TRPM3 cells with a Coll.luc-containing lentivirus and maintained the cells in a medium containing tetracycline to induce the expression of TRPM3 expression. Cells were cultured in the serum-reduced medium for 24 h and preincubated with the potent and selective CBP/p300 histone acetyltransferase inhibitor A485 (Figure 1B). The results show that the presence of A485 attenuated the TRPM3-mediated activation of AP-1. AP-1 activity was reduced by more than 80% when we incubated T-REx-TRPM3 cells with the histone acetyltransferase inhibitor A485 (Figure 2B). Next, we used the compound JQ1 (Figure 1D), a thieno-triazolo-1,4-diazepine that functions as an inhibitor of BET proteins. JQ1 binds competitively to the bromodomains of BET proteins and displaces these proteins from chromatin [18]. The modular structure of two BET proteins, BRD3 and BRD4, is depicted in Figure 1C. The presence of the bromodomain inhibitor JQ1 in the culture medium resulted in an 80% reduction of AP-1 activity in stimulated T-REx-TRPM3 cells. Thus, stimulation of AP-1 following TRPM3 activation is controlled by the coactivators CBP/p300 and the BET acetyl-lysine binding proteins.

### 2.2. Inhibitors of CBP/p300 and BET Proteins Reduce the TRPM3-Mediated Stimulation of the Interleukin-8 (IL-8) Promoter

**Figure 3:**
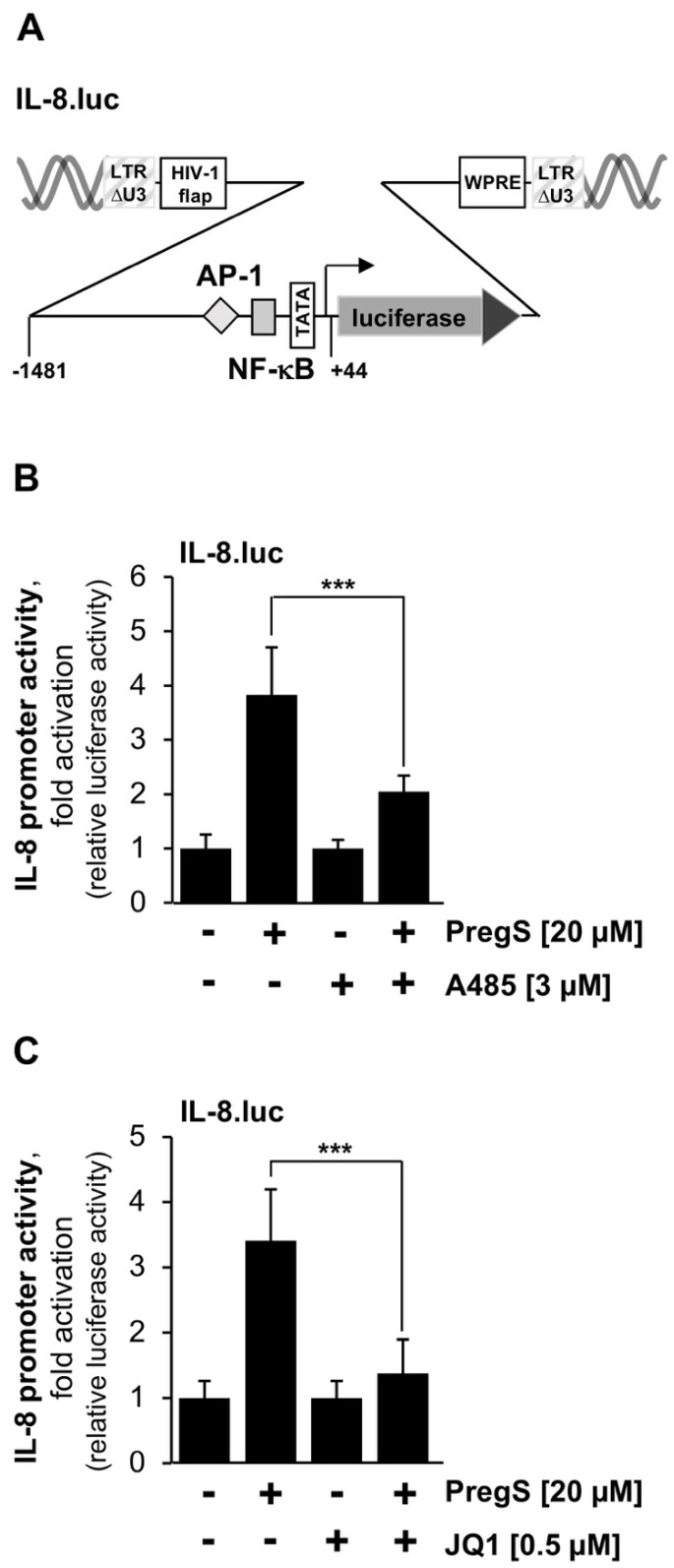
Pharmacological inhibition of CBP/p300 and BET proteins attenuates interleukin-8 promoter activity following stimulation of TRPM3 channels. (**A**) Provirus containing the IL-8.luc reporter gene, encompassing IL-8 gene sequences from −1481 to +44. (**B**,**C**) T-REx-TRPM3 cells were infected with a recombinant lentivirus containing the IL-8.luc reporter gene. The cells were treated, stimulated, harvested, and analyzed as described in the legend in Figure 2. Data shown are mean +/− SD of four (**B**) or three (**C**) independent experiments performed in quadruplicate (*** *p* < 0.001).

To confirm the observation that the TRPM3-mediated stimulation of AP-1 is controlled by CBP/p300 and BET proteins, we analyzed the transcription of a recently identified target gene of AP-1, the gene encoding the cytokine interleukin-8 (IL-8). Stimulation of TRPM3 channels activates IL-8 gene transcription and required the AP-1 binding found in the proximal IL-8 gene promoter [7]. We integrated the IL-8.luc reporter gene into the chromatin of T-REx-TRPM3 cells. Transcription of the reporter gene was driven by the IL-8 promoter (sequence −1481 to +44) (Figure 3A). Tetracycline-treated T-REx-TRPM3 cells were stimulated with pregnenolone sulfate. In addition, we added either A485 or JQ1 to the culture medium. Inhibition of CBP/p300 activity reduced IL-8 promoter-controlled gene transcription by 61% (A485, Figure 3B), whereas incubation of T-REx-TRPM3 cells with the compound JQ1 reduced IL-8 promoter activity by more than 80% (JQ1, Figure 3C).

### 2.3. The Transcriptional Activation Potential of the Transcription Factor c-Fos Was Reduced by CBP/p300 and BET Protein Inhibitors in Stimulated T-REx-TRPM3 Cells

**Figure 4:**
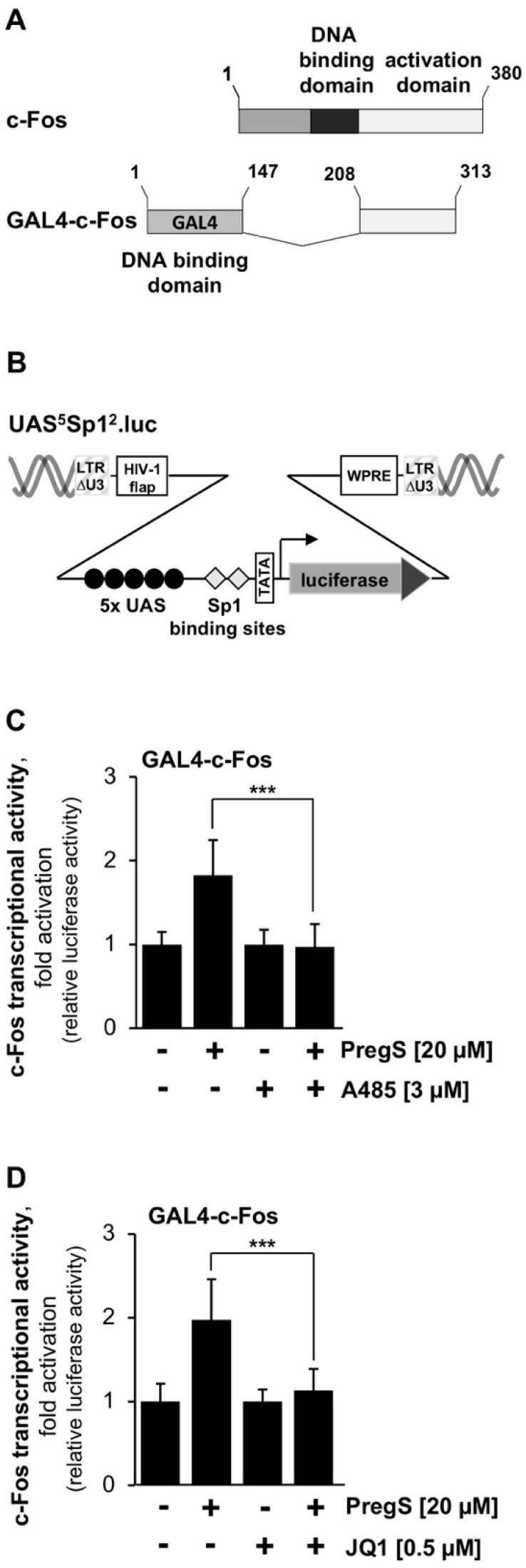
Pharmacological inhibition of CBP/p300 and BET proteins attenuates TRPM3-induced upregulation of the transcriptional activation potential of c-Fos. (**A**) The modular structure of the GAL4-c-Fos fusion protein. (**B**) Provirus that encodes the GAL4-responsive reporter gene UAS^5^Sp1^2^.luc. (**C**,**D**) T-REx-TRPM3 cells were infected with a UAS^5^Sp1^2^.luc containing lentivirus. T-REx-TRPM3 cells were infected with a second lentivirus that encoded for the GAL4-c-Fos protein. The cells were maintained in the serum-reduced medium in the presence of tetracycline for 24 h. T-REx-TRPM3 cells were stimulated with pregnenolone sulfate (PregS, 20 μM) for 24 h in the presence or absence of A485 (3 μM) (**C**), or JQ1 (0.5 μM) (**D**). Cells were harvested and analyzed as described in the legend to Figure 2 ((**C**), ***n*** = 6; (**D**), ***n*** = 3; *** *p* < 0.001).

Proteins of the transcription factor families Jun, Fos, and ATF form the dimeric AP-1 complex. These proteins possess a leucine zipper domain for dimerization. Stimulation of TRPM3 channels activates the c-Fos promoter and increases the biological activity of c-Fos [3]. We investigated whether the presence of either the CBP/p300 inhibitor A485 or the BET protein inhibitor JQ1 changes the biological activity of c-Fos. The transcriptional activation potential, i.e., the biological activity of c-Fos, was measured in T-REx-TRPM3 cells expressing a GAL4-c-Fos fusion protein (Figure 4A). The DNA interaction domain of GAL4 binds to a reporter gene that contained binding sites for GAL4 (UAS, upstream regulatory sequence) in its regulatory region (Figure 4B). T-REx-TRPM3 cells were infected with a lentivirus containing the reporter gene UAS^5^Sp1^2^.luc. T-REx-TRPM3 cells were additionally infected with a lentivirus encoding the GAL4-c-Fos fusion protein. The infected cells were incubated in serum-reduced and tetracycline-supplemented medium and stimulated with pregnenolone sulfate. The compounds A485 or JQ1 were added as indicated. Figure 4C shows that inhibition of CBP/p300 acetyltransferase activity by A485 completely blocked the biological activity of c-Fos. Inhibition of BET protein interaction with acetylated lysine residues reduced the transcriptional activation potential of c-Fos by 76% (JQ1, Figure 4D).

### 2.4. The Transcriptional Activation Potential of the Ternary Complex Factor Elk-1 Is Reduced in the Presence of Either CBP/p300 or BET Protein Inhibitors in Stimulated T-REx-TRPM3 Cells

**Figure 5:**
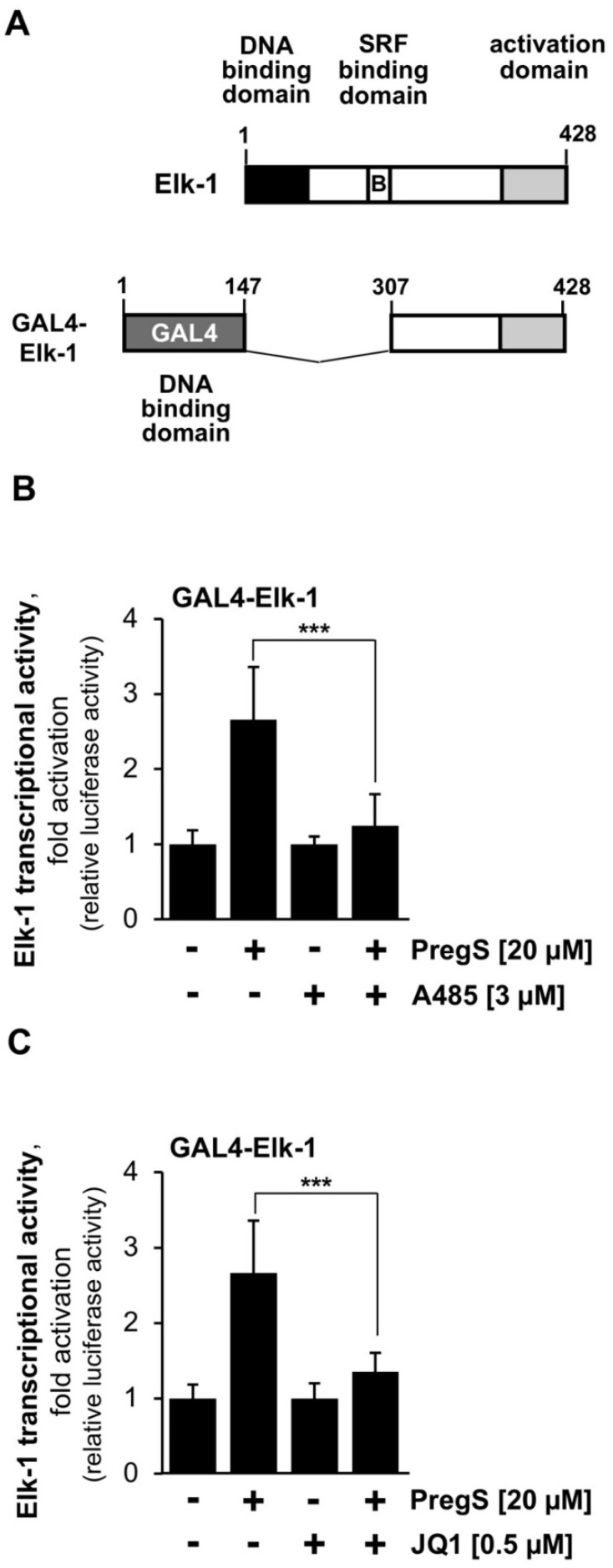
CBP/p300 and BET inhibitors reduce the transcriptional activation potential of Elk-1 in stimulated T-REx-TRPM3 cells. (**A**) Domain structure of GAL4-Elk-1 (**B**,**C**) T-REx-TRPM3 cells were infected with a lentivirus containing the UAS^5^Sp1^2^.luc reporter gene. Cells were infected with a second lentivirus that encoded for the GAL4-Elk-1 fusion protein. T-REx-TRPM3 cells were maintained in serum-reduced and tetracyclin-supplemented medium for 24 h. Stimulation was performed with pregnenolone sulfate (PregS, 20 μM) for 24 h. Addition of either A485 (3 μM) (**B**) or JQ1 (0.5 μM) (**C**) is indicated. Cells were harvested and analyzed as described in the legend to Figure 2 (***n*** = 3; *** *p* < 0.001).

The ternary complex factor Elk-1 controlls c-Fos expression and in this way regulates AP-1 activity [2,6]. We therefore investigated whether the biological activity of Elk-1 is changed in the presence of CBP/p300 or the BET protein inhibitors. We used a GAL4-Elk-1 fusion protein (Figure 5A) to measure the transcriptional activation potential of Elk-1. Elk-1 activity is regulated by phosphorylation, mediated by a phosphorylation-responsive activation domain within the Elk-1 protein. This activation domain is fused to the DNA binding domain of GAL4. GAL4-Elk-1 was expressed in T-REx-TRPM3 cells containing the GAL4-responsive reporter gene UAS^5^Sp1^2^.luc. Figure 5B shows that TRPM3-mediated activation of Elk-1 was reduced by 88% when cells were incubated with A485. The transcriptional activation potential of Elk-1 was reduced by 76% with compound JQ1 in stimulated T-REx-TRPM3 cells (Figure 5C).

### 2.5. Measurement of cAMP Response Element-Mediated-Mediated Transcription Using a CRE-Containing Reporter Gene and a CREB Mutant

**Figure 6:**
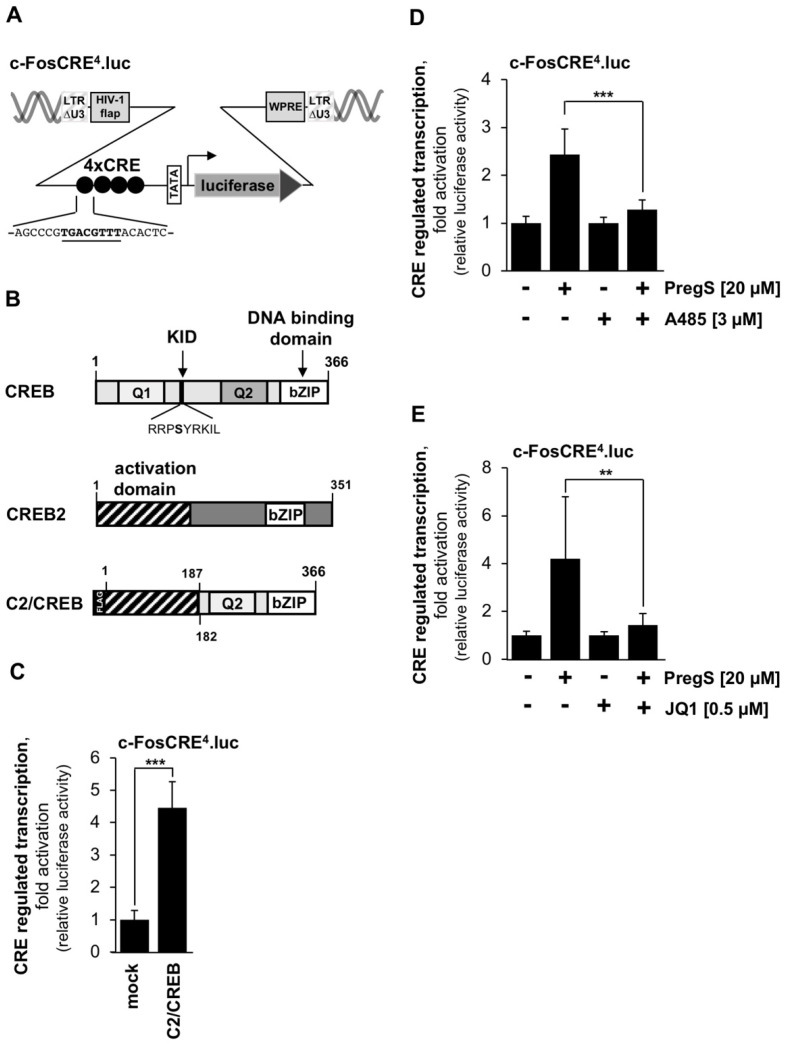
Pharmacological inhibition of CBP/p300 and BET bromodomain proteins attenuates TRPM3-induced activation of CREB. (**A**) Provirus containing the c-FosCRE^4^.luc gene. The sequence of the CRE is shown. (**B**) Domain structures of CREB, CREB2 and C2/CREB. KID, kinase inducible domain; Q1, Q2, glutamine-rich activation domains of CREB. (**C**) T-REx-TRPM3 cells were infected with a lentivirus encoding either C2/CREB or β-galactosidase (mock). Additionally, lentiviral gene transfer was used to integrate the c-FosCRE^4^.luc reporter gene into the chromatin. Infected T-REx-TRPM3 cells were maintained for 3 days. Cell extracts were prepared, luciferase activities were determined and protein concentrations were measured. The protein concentration were used to normalize the luciferase activities (***n*** = 4; *** *p* < 0.001). (**D**,**E**) T-REx-TRPM3 cells containing a chromatin-embedded c-FosCRE^4^.luc reporter gene were maintained in serum-reduced and tetracycline-supplemented medium for 24 h. Stimulation was performed with pregnenolone sulfate for 24 h. Additon of either A485 (3 μM) (**D**) or JQ1 (0.5 μM) (**E**) to the culture medium is indicated. T-REx-TRPM3 cells were harvested and analyzed as described in the legend to Figure 2 (***n*** = 3; ** *p* < 0.01; *** *p* < 0.001).

CBP was discovered as a co-activator of CREB (cAMP response element (CRE) binding protein) that regulates CREB-mediated transcription. Soon after, a similar activity was detected for p300 [19,20]. We therefore sought to determine whether stimulation of TRPM3 activates CREB, and if this is the case, whether CBP and p300 control transcription via CRE following stimulation of TRPM3 channels. To measure CRE-mediated transcription we used the c-FosCRE^4^.luc reporter gene, which contains four copies of CRE derived from the c-Fos gene in its regulatory region (Figure 6A). To demonstrate the functionality of this reporter gene, a constitutively active CREB mutant, C2/CREB, was expressed in T-REx-TRPM3 cells. The domain structures of CREB, CREB2 and C2/CREB are shown in Figure 6B. C2/CREB contains the phosphorylation-independent activation domain of CREB2. Expression of C2/CREB in T-REx-TRPM3 cells resulted in a significant upregulation of CRE-mediated transcription (Figure 6C), indicating that the reporter gene functions very well as a sensor for CRE/CREB-regulated transcription.

### 2.6. CBP/p300 and BET Proteins Control the Activation of CREB-Regulated Gene Transcription in Stimulated T-REx-TRPM3 Cells

We next analyzed whether stimulation of TRPM3 channels leads to increased transcription of the CRE-containing c-FosCRE^4^.luc reporter gene. Figure 6D,E show that stimulation of TRPM3 channels induced transcription of a CRE-containing reporter gene. This stimulation was impaired when we cultured the cells in the presence of A485 (Figure 6D) or JQ1 (Figure 6E). CRE-mediated transcription was reduced by more than 70% in the presence of the CBP/p300 inhibitor (Figure 6D), whereas incubation of the cells with the BET inhibitor JQ1 inhibited the CREB-regulated gene transcription by 87% (Figure 6E).

## 3. Discussion

Transcriptional activation by stimulus-responsive transcription factors requires access for these proteins to their DNA binding sites. DNA is embedded in a chromatin environment that may have an open or a compact configuration. Transcriptional co-regulators modify the chromatin structure and can act as a bridge between the basic transcriptional apparatus and DNA-bound transcription factors. In addition, the cofactors can cause specific local changes in the chromatin structure. These changes often include posttranscriptional modifications of histones, the proteins that form the core of the nucleosome. One important modification is the acetylation of histones, catalyzed by acetyltransferases. Hyperacetylation of histones is frequently observed in transcriptionally active genes. Acetylation of lysine residues reduces the positive charges of histone proteins, resulting in a lower affinity for acidic DNA and an altered nucleosomal structure. Histone acetylation thus facilitates transcription factors´access to their DNA binding sites. Furthermore, several histone acetyltransferases interact with RNA polymerase II or with proteins of the basic transcriptional apparatus, establishing a causal link between histone acetylation and transcriptional activation [8,9]. Stimulation of TRPM3 Ca^2+^ channels activate transcription mediated by AP-1, c-Fos, c-Jun, Egr-1, and Elk-1. In this study, we investigated whether the activity of transcriptional coactivators and modifications of the chromatin structure are essential for TRPM3-mediated transcription.

Two of the most promiscuous acetyltransferases are the proteins CBP and p300. Both proteins interact with numerous transcriptional activator proteins such as CREB, p53, c-Jun, nuclear hormone receptors, NF-κB and others [13,14,15]. These interactions regulate biological functions such as cellular growth, proliferation, apoptosis, senescence, and differentiation. CBP/p300 proteins are acetyltransferases and their enzymatic activity contributes to chromatin-mediated transcriptional activation [15,21,22]. CBP/p300 proteins are considered global transcriptional co-activators that acetylate lysine residues in histones H2A, H2B, H3 (in particular H3K18, H3K27, and H3K56), and H4, and in non-histone proteins such as p53. In addition, CBP and p300 have been shown to promote the recruitment of RNAase polymerase II to enhancers, indicating that CBP/p300-catalyzed acetylation is essential for transcriptional initiation of enhancer-controlled gene transcription [23].

To selectively inhibit CBP/p300 activity, we used the potent and selective CBP/p300 catalytic inhibitor A485, a spirooxazolidinedione derivative. In contrast, the often-used compound C646 showed off-target inhibitory effects [24]. A quantitative acetylomics study showed that treatment of cells with A485 reduced the acetylation pattern at approximately 1000 acetyl-lysine sites [25]. The co-crystal structure of the p300 catalytic active site together with the A485 molecule showed that A485 competes with acetyl-CoA. A485 did not inhibit the activities of histone acetyltransferases such as PCAF and others [26]. Functional assays revealed that this compound inhibited tumor growth [27,28]. The results of this study showed that incubation of cells with the compound A485 significantly decreased the activation of AP-1 in stimulated T-REx-TRPM3 cells, indicating that CBP/p300 proteins are involved in the signaling cascade linking TRPM3 Ca^2+^ channels to transcription of AP-1-responsive genes. Recently, the expression of the cytokine interleukin-8 was shown to be regulated by AP-1 [7]. Accordingly, TRPM3-induced activation of the IL-8 promoter was reduced in the presence of the CBP/p300 inhibitor A485. These results are in agreement with the observation that pro-inflammatory gene expression was inhibited by the p300 inhibitor C646 [28]. Moreover, in this study, we demonstrated for the first time that TRPM3 stimulation activates CREB and that this activation also requires CBP/p300. Finally, analysis of the transcriptional activation potential of c-Fos and Elk-1 revealed that upregulation of the transcriptional activities of these transcription factors requires CBP/p300. This study supports previous investigations showing that stimulus-activated transcription of target genes involving AP-1, CREB, or Elk-1 requires CBP and/or p300 activity [19,29].

While histone acetyltransferases are considered “writers” of posttranslational histone modifications, bromodomain-containing proteins are “readers” that bind to acetylated histone molecules [11]. Bromodomains are folding units comprising approximately 110 amino acids that are found in 46 human proteins that recognize acetylated lysine residues via a central hydrophobic pocket. Bromodomain inhibitors form hydrogen bonds with a conserved asparagine residue of the BET bromodomain, disrupting the interaction between acetylated lysines and BET bromodomains. By this means, the binding mode of acetyl-lysine is mimicked. CBP and p300 belong to the bromodomain proteins, as shown in Figure 1A. Acetylation of lysine residues of histone proteins is catalyzed by CBP and p300, thus creating binding sites for their bromodomain, which can stabilize chromatin interaction via a positive feedback loop. The BET proteins, which include BRD2, BRD3, BRD4, and testis-specific BRDT, are involved in the regulation of epigenetic gene transcription by regulating chromatin opening, recruitment of transcription factors, and co-activators. While BET proteins have been shown to be involved in transcriptional control, the role of bromodomains in other bromodomain proteins in regulating gene transcription has not been clearly defined [30]. Experiments with I-CBP112, a CBP/p300 bromodomain inhibitor, showed that this compound has only marginal effects on gene transcription [31]. To assess the importance of bromodomain-containing proteins in regulating transcription after stimulation of TRPM3 channels, we chose the highly potent and selective pan-BET inhibitor JQ1. This compound has a rather weak binding activity to CBP/p300, but binds competitively to bromodomains of all BET proteins [18,32]. Treatment with JQ1 displaces BET proteins from nuclear chromatin and thus regulates the expression of numerous target genes, including P-TEFb [18,33].

The results of this study showed that inhibition of bromodomain binding to acetylated lysine attenuated TRPM3-mediated activation of the transcription factors AP-1 and CREB. Moreover, TRPM3-induced activation of interleukin-8 expression was attenuated by the pan-BET inhibitor JQ1, supporting the view that BET proteins regulate inflammatory cytokine gene transcription [34,35]. Our data complement a study investigating the role of bromodomain proteins in endothelial cell inflammation [36], showing that treatment with JQ1 caused a reduction in interleukin-8 mRNA and protein levels. We also demonstrated that the biological activity of c-Fos and Elk-1 in TRPM3-stimulated cells was attenuated in the presence of JQ1. Thus, we have identified for the first time bromodomain proteins as regulators of TRPM3-induced gene transcription.

Our results showed that stimulation of TRPM3 channels activated gene transcription involving CBP/p300 and BET proteins. TRPM3 stimulation triggers an influx of Ca^2+^ ions into the cells and activation of the protein kinase ERK1/2. An interesting question is whether the signaling cascade has an impact on the epigenetic regulation of transcription via post-translational modifications of epigenetic regulators. These regulators, like stimulus-responsive transcription factors, could act as an interface between extracellular signals and the transcription of selected genes. CBP is a substrate for protein kinases. In addition, activation of ERK1/2 and CaMKIV has been shown to be important in triggering neural activity-mediated gene transcription via NMDA receptors [37]. Two stimulus-responsive transcriptional activation domains have been identified in the CBP molecule [38]. Therefore, we hypothesize that different stimuli that trigger a similar signaling cascade in the cell may lead to similar activation of CBP/p300. The regulation of BET proteins by phosphorylation remains relatively unexplored. The BET protein BRD4 is a phosphoprotein and phosphorylation may be regulated by IL-6/IL-8-induced signaling [39]. However, the binding of BET proteins to acetylated lysine residues may be sufficient to induce their biological activities. With the specific compounds A485 and JQ1 at hand, future studies may elucidate the link between cellular stimulation and epigenetic regulation via CBP/p300 and BET proteins.

## 4. Materials and Methods

### 4.1. Cell Culture and Reagents

HEK293 cells containing the human TRPM3 coding region under the control of a tetracycline-responsive promoter (T-REx-TRPM3 cells) were kindly provided by David Beech and Yasser Majeed, University of Leeds, Leeds, UK, and cultured as described [40]. TRPM3 expression was induced by adding tetracycline (1 μg/mL, Sigma-Aldrich #T7680, dissolved in water) to the culture medium containing 0.05% fetal bovine serum for 24 h prior to the stimulation with pregnenolone sulfate (PubChem CID: 105074). Stimulation with pregnenolone sulfate (20 μM, Sigma #P162, dissolved in DMSO) was performed for 24 h in DMEM containing 0.05% fetal bovine serum. The selective CBP/p300 inhibitor A485 (A485: PubChem ID 118958122).

((1*R*)-*N*-[(4-Fluorophenyl)methyl]-2,3-dihydro-5-[[(methylamino)carbonyl]amino]-2′,4′-dioxo-*N*-[(1*S*)-2,2,2-trifluoro-1-methylethyl]spiro [1*H*-indene-1,5′-oxazolidine]-3′-acetamide) was purchased from Tocris (Bristol, UK, #6387), dissolved in DMSO and used at a final concentration of 3 μM. The BET bromodomain inhibitor JQ1 (PubChem ID 46907787),(6*S*)-4-(4-Chlorophenyl)-2,3,9-trimethyl-6*H*-thieno [3,2-*f*][1,2,4]triazolo [4,3-*a*][1,4]diazepine-6-acetic acid 1,1-dimethylethyl ester) was purchased from Adooq Bioscience (Irvine, CA, USA, #A12729), dissolved in DMSO and used at a final concentration of 0.5 μM. The chemical structures of A485 and JQ1 are depicted in Figure 1B,D, respectively.

### 4.2. Lentiviral Gene Transfer

The lentiviral transfer vectors pFUW-C2/CREB, pFUW-GAL4-c-Fos, and pFUW-GAL4-Elk-1 have been described elsewhere [41,42]. The viral particles were produced by triple transfection of HEK293-TN cells with the packing plasmid pCMVΔR8.91 (5 μg/plate), plasmid pCMV-G (2.3 μg/plate) encoding the vesicular stomatitis virus glycoprotein, and the lentiviral transfer vector (6.6 μg/plate) using the calcium phosphate transfection method as described [2]. Cells were incubated overnight with the calcium phosphate-DNA precipitate in the presence of chloroquine (25 μM). Cells were washed the next day with phosphate-buffered saline and maintained for 72 h in DMEM medium containing 10% serum. Viral supernatants were harvested, centrifuged, and filtered through a 0.45 µM filter. Final viral stocks were supplemented with polybrene at a final concentration of 8 μg/mL.

### 4.3. Reporter Assays

The lentiviral transfer vectors pFWColl.luc, pFWIL-8.luc, pFWUAS^5^Sp1^2^.luc, and pFWc-FosCRE^4^.luc have been described elsewhere [7,16,42]. T-REx-TRPM3 cells were infected with recombinant lentiviruses containing promoter/luciferase reporter genes. Following stimulation, cell extracts were prepared using reporter lysis buffer (Promega, Mannheim, Germany) and analyzed for luciferase activities. Luciferase activities of the extracts were measured using a luminometer (Berthold Detection Systems, Huntsville, AL, USA). Light units were normalized to the protein concentration of the extracts, determined with a BCA protein assay kit.

### 4.4. Statistics

Statistical analyses were done by using the two-tailed Student’s *t*-test. Statistical probability is expressed as * *p* < 0.05, ** *p* < 0.01, and *** *p* < 0.001. Values were considered significant when *p* < 0.05.

## 5. Conclusions

**Figure 7:**
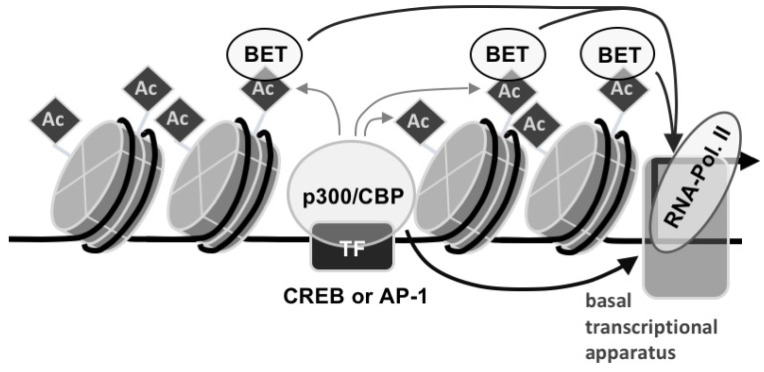
Regulation of epigenetic gene transcription by CBP/p300 and BET bromodomain proteins.

In recent years, we have described in detail the intracellular signaling cascade triggered by TRPM3 Ca^2+^ channel stimulation. The studies have shown that Ca^2+^ ions, protein kinases, and calcineurin link TRPM3 channel activation to transcription factor stimulation. The present study provides evidence that epigenetic modulators are required in addition to the sequence-specific binding of transcription factors to DNA. CBP/p300 are histone acetylases that acetylate lysine residues of histones and in this way induce an open chromatin structure. Our pharmacological analysis revealed that the coactivators CBP and p300 are essential for connecting TRPM3 stimulation with gene transcription. Acetylated lysine residues function as recognition motives for epigenetic BET proteins that further regulate epigenetic-controlled transcription via the recruitment of transcription factors, co-activators, and chromatin-organizing proteins. The fact that inhibition of BET protein interaction with acetyl-lysine residues attenuates TRPM3-mediated gene transcription indicates that BET proteins are important epigenetic regulatory proteins of TRPM3-mediated transcription. Based on these data, we propose a chromatin-based signaling cascade, involving DNA-bound stimulus-responsive transcription factors, recruitment of CBP/p300, acetylation of histones, and subsequent recruitment of the BET proteins to the transcription unit (Figure 7). Future studies will show whether the regulation of other biological functions of TRPM3 also requires CBP/p300 and BET proteins.

Transcription factors such as CREB and AP-1 bind to specific DNA binding sites and recruit the coactivator and histone acetyltransferase proteins CBP and p300. These proteins catalyze the acetylation of histones, and by this means generate an open chromatin architecture. CBP/p300 also acts as a bridge to the basal transcriptional apparatus to support transcriptional activation. BET proteins bind to acetylated lysine residues in histone proteins and recruit coactivators and epigenetic regulators required for transcription.

## Data Availability

Data is contained within the article.
